# Peritoneal Scintigraphy confirming pleural-peritoneal fistula

**DOI:** 10.1007/s40620-023-01729-2

**Published:** 2023-08-03

**Authors:** Erik Lawrence Lum, Michelle Hwang, Ali Salavati, Jenny In Shen

**Affiliations:** 1 Division Nephrology, Department of Medicine, UCLA David Geffen School of Medicine, Connie Frank Kidney Transplant Clinic, 200 Medical Plaza, Ste 565, Los Angeles, CA 90095 USA; 2 Ahmanson Translational Theranostics Division, David Geffen School of Medicine, UCLA, Los Angeles, CA USA; 3 The Lundquist Institute at Harbor-UCLA Medical Center, Los Angeles, USA

**Keywords:** ESRD, Pleural-peritoneal fistula, FSGS, Peritoneal dialysis, Scintigraphy

**Fig. 1:**
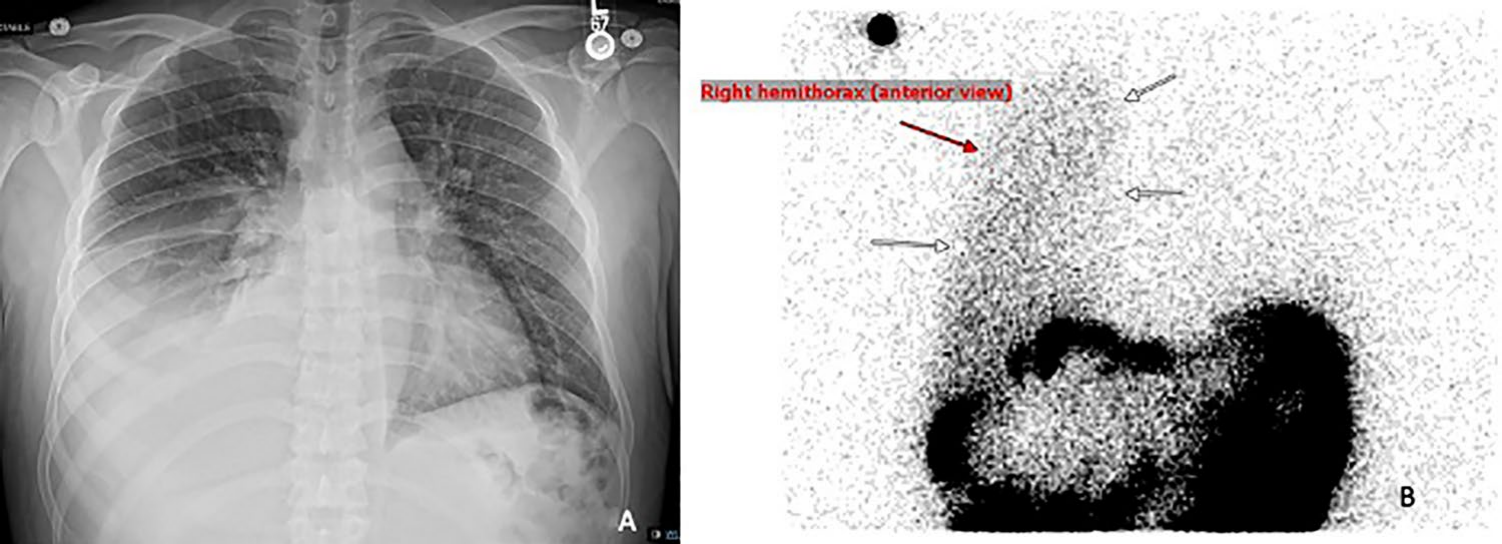
**a** chest X-ray. **b** Scintigraphy images indicating pleural-peritoneal fistula

A 32-year-old male with kidney failure secondary to focal segmental glomerulosclerosis who had been on peritoneal dialysis for 15 months, presented to the emergency room with shortness of breath and bilateral lower extremity edema worsening over the past 7 days. He reported full adherence with peritoneal dialysis and multiple hospitalizations in the past 6 months for recurrent right pleural effusion and volume overload. Physical examination showed a temperature of 37 °C, blood pressure of 120/67 mmHg, oxygen saturation of 89% on room air, a peritoneal dialysis catheter in the right lower quadrant and decreased breath sounds with dullness to percussion of the right lung. A chest X-ray showed a large right pleural effusion (Fig. 1A). He declined thoracentesis due to previous pneumothorax complication. Given concerns for peritoneal leak he underwent radionucleotide scintigraphy with intraperitoneal injection of technetium-99 m sulfur colloid [1]. Delayed images after injection demonstrated radiotracer uptake in the right pleural cavity, confirming evidence of a right pleural-peritoneal communication (Fig. 1B). He agreed to undergo therapeutic thoracentesis and was transitioned to maintenance hemodialysis, which he remains on while awaiting deceased donor kidney transplantation.
